# Carbon nanotubes (CNT)-loaded ginsenosides Rb3 suppresses the PD-1/PD-L1 pathway in triple-negative breast cancer

**DOI:** 10.18632/aging.203131

**Published:** 2021-06-10

**Authors:** Xiao Luo, Hui Wang, Degang Ji

**Affiliations:** 1 Department of Breast Surgery, China-Japan Union Hospital of Jilin University, Changchun 130033, Jilin, China; 2 Department of Ultrasound, China-Japan Union Hospital of Jilin University, Changchun 130033, Jilin, China; 3 Department of Hepatobiliary Pancreatic Surgery, China-Japan Union Hospital of Jilin University, Changchun 130033, Jilin, China

**Keywords:** triple-negative breast cancer, progression, CNTs, Rg3, PD-1/PD-L1 axis

## Abstract

Carbon nanotubes (CNTs), as advanced nanotechnology with specific properties and structures, have presented practical drug delivery properties. Ginsenoside Rg3 is a component of puffed ginseng and demonstrates anti-cancer activities. To explore the effect of CNTs-loaded Rg3 (Rg3-CNT) on the PD-1/PD-L1 signaling and the development of triple-negative breast cancer (TNBC). Our data revealed that Rg3 inhibited the cell viability of TNBC cells, in which Rg3-CNT further enhanced this effect in the system. Similarly, the colony formation of TNBC cells was decreased by Rg3, while Rg3-CNT could reinforce its effect in the cells. Besides, the treatment of Rg3 induced apoptosis of TNBC cells, in which Rg3-CNT treatment further increased the phenotype in the cells. Remarkably, Rg3-CNT, but not Rg3, attenuated PD-L1 expression in TNBC cells. Rg3-CNT decreased the PD-L1 upregulation induced by interferon-γ (IFN-γ) in breast cancer cells. Importantly, Rg3-CNT was able to reduce PD-1 expression in activated T cells. Specifically, Rg3-CNT reduced the PD-1/PD-L1 axis in a T cell/triple-negative TNBC cell co-culture system. Moreover, the levels of IFN-γ, interleukins-2 (IL-2), interleukins-9 (IL-9), interleukins-10 (IL-10), interleukins-22 (IL-22), and interleukins-23 (IL-23) were significantly stimulated in the activated T cells, while the treatment of Rg3-CNT could reverse these phenotypes in the cells. Rg3-CNT attenuated the TNBC cell growth *in vivo.* The Rg3-CNT improved the anti-cancer effect of Rg3 toward TNBC by inhibiting the PD-1/PD-L1 axis. Our finding provides new insights into the mechanism by which Rg3-CNT attenuates the development of TNBC. Rg3-CNT may be applied as the potential therapeutic strategy for immunotherapy of TNBC.

## INTRODUCTION

Triple-negative breast cancer (TNBC) is one of the most common and lethal cancers in women globally [1]. It is still the second primary risk for cancer-correlated death in women despite the advancement of various treatments [2]. The adjuvant therapy, radical surgery, and early diagnosis enhance patients' survival rates and prognosis incidence with breast cancer, but mortality incidence is still unsatisfactory [3]. The development of effective therapeutic strategies for breast cancer treatment is urgently required [4]. Moreover, immune evasion is a significant challenge for anti-cancer immunotherapy [5, 6]. Cancer cells are able to expand a massive magazine of pathways and molecules to evade immune monitoring and counter the host T cell cytotoxic impact, including by regulating the immune checkpoints [7, 8]. Among the immune checkpoints, PD-1/PD-L1 axis shows the fundamental function, and thereby targeting PD-1/PD-L1 signaling has been recognized as a promising choice to overwhelm exhaustion of T cells and extract efficient anti-cancer immune answers [9–11]. However, the advancement in the exploration of therapeutic treatment for targeting the PD-1/PD-L1 pathway remains limited.

Nanomaterials with the sizes ranging from 1 nm-100 nm present biological, chemical, and physical characteristics [12, 13]. It has demonstrated significant potential in diagnostics, imaging, drug delivery, and other medical applications [12, 13]. Carbon nanotubes (CNTs) serve as the carbon allotrope and various imaginary cut lines lead to various kinds of CNTs with different structures: such as chiral, armchair, and zigzag [14, 15]. CNTs show multiple advanced properties as the drug carrier, including large surface area, thermal properties, remarkably penetrating capability on the cell membrane, and electronic properties [16]. CNTs, containing cylindrical nanostructure of carbon allotropes, have attained enormous recognition in biomedical employment due to the individual properties and structures, such as size stability on the nanoscale, generous surface chemical functionalities, extensive surface areas, large aspect ratios [17–19]. Especially, CNTs are engaging as mediators and carriers for the treatment of cancers. Given their advanced functionalization, CNTs have been applied as nanocarriers for anti-tumor therapies, such as RNA/DNA aptamers, oligonucleotides, small-interfering RNA, paclitaxel, cisplatin, carboplatin, doxorubicin [14]. Moreover, Ginsenoside Rg3 is a component obtained from puffed ginseng and presented anti-tumor activity in breast cancer [20, 21]. Rg3 can induce an inhibitory effect on cancer development by affecting different processes and pathways, such as metastasis, DNA damage, epigenetic modification, cancer stemness, drug resistance, PI3K/AKT signaling [22–27], but its correlation with PD-L1 is poorly understood. Meanwhile, it has been identified that two other gradients of ginsenoside, termed Rb1 and Rg1, can be carried by CNTs and induce an inhibitory effect on cancer development [28]. However, the role of CNTs-loaded Rg3 (Rg3-CNT) in the regulation of breast cancer remains elusive.

In the present investigation, we were interested in the functions of Rg3-CNT in TNBC. We reported the new roles of Rg3-CNT in improving the anti-cancer effect of Rg3 toward TNBC by inhibiting PD-1/PD-L1 axis.

## RESULTS

### Rg3-CNT represses proliferation and increases apoptosis of TNBC cells

**Figure 1:**
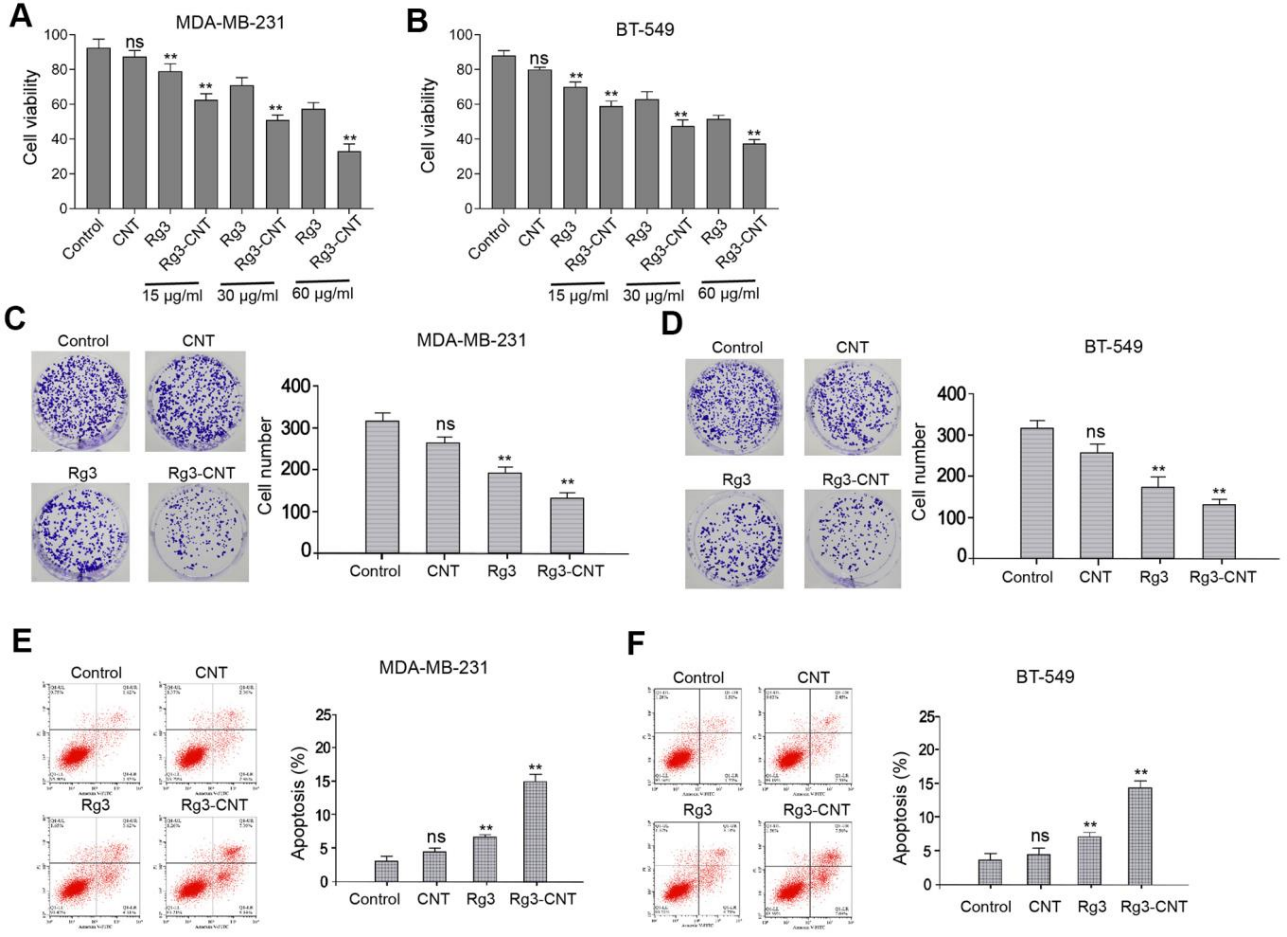
**Rg3-CNT inhibits proliferation and induces apoptosis of TNBC cells.** (**A**–**F**) The MDA-MB-231 and BT-549 cells were treated with CNT, Rg3, or Rg3-CNT. (**A**, **B**) The cell viability was measured by the MTT assays in the cells. (**C**, **D**) The cell proliferation was analyzed by the colony formation assays in the cells. (**E**, **F**) The cell apoptosis was assessed by flow cytometry analysis in the cells. Data are presented as mean ± SD. Statistic significant differences were indicated: ns no significance, ** *P* < 0.01.

To measure the functions of Rg3-CNT in TNBC, MDA-MB-231 and BT-549 cells were treated with CNT, Rg3, or Rg3-CNT. Our data showed that the treatment of Rg3, but not CNT, inhibited the cell viabilities of MDA-MB-231 and BT-549 cells, in which Rg3-CNT further enhanced this effect in the system (Figure 1A, 1B). Similarly, colony formation of MDA-MB-231 and BT-549 cells was reduced by Rg3 while Rg3-CNT could reinforce its effect in the cells (Figure 1C, 1D). Moreover, the treatment of Rg3 induced apoptosis of MDA-MB-231 and BT-549 cells, in which Rg3-CNT treatment further increased the phenotype in the cells (Figure 1E, 1F). The similar results were observed in MDA-MB-468 cell lines (Supplementary Figure 1), suggesting that Rg3-CNT inhibits proliferation and induces apoptosis of TNBC cells.

### Rg3-CNT decreases PD-L1 expression in TNBC cells

**Figure 2:**
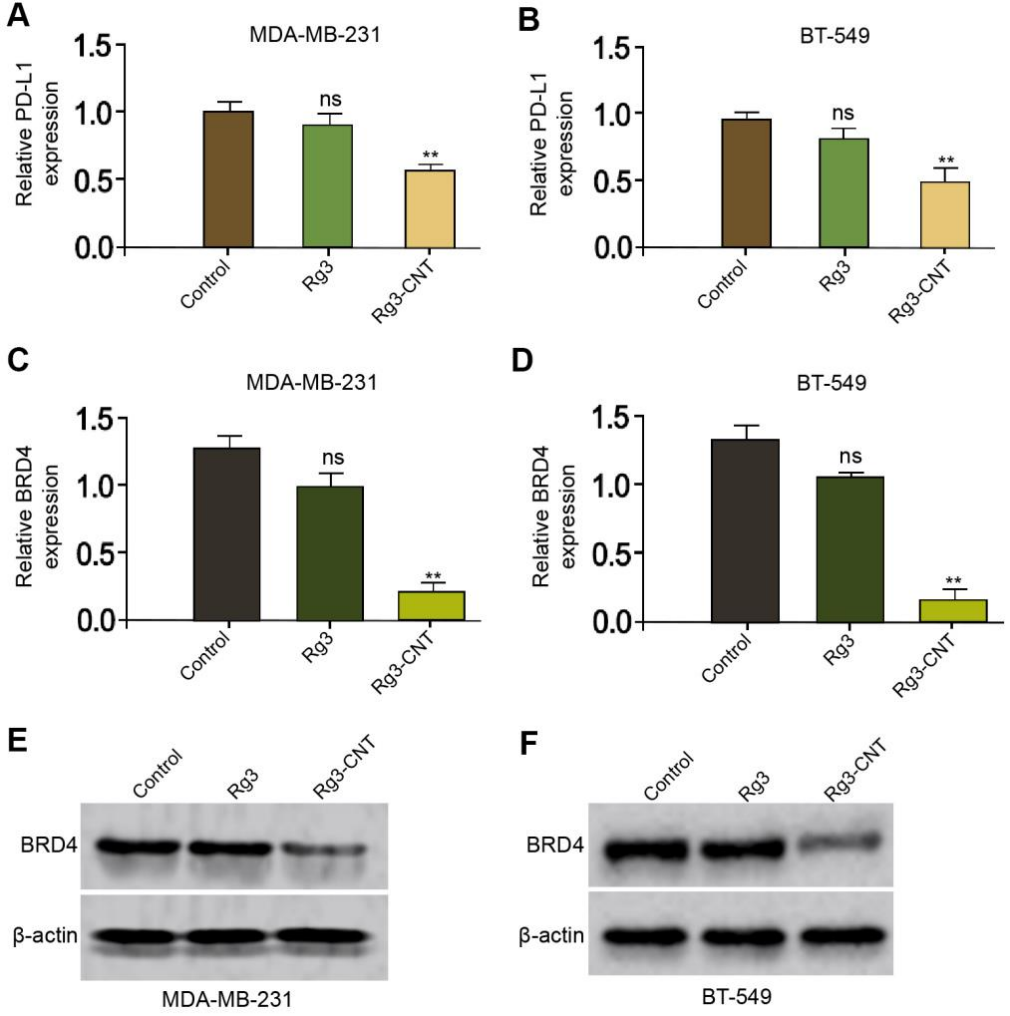
**Rg3-CNT decreases PD-L1 expression in TNBC cells.** (**A**–**E**) The MDA-MB-231 and BT-549 cells were treated with Rg3 (60 μg/ml) or Rg3-CNT (60 μg/ml). (**A**, **B**) The expression of PD-L1 was analyzed by fluorescence-activated cell sorting (FACS) in the cells. (**C**, **D**) The mRNA expression of BRD4 was measured by qPCR in the cells. (**E**, **F**) The protein expression of BRD4 was tested by Western blot analysis in the cells. Data are presented as mean ± SD. Statistic significant differences were indicated: ns no significance, ** *P* < 0.01.

It has been reported that Rg3 inhibits cisplatin resistance by repressing PD-L1 lung cancer and the inhibition of BRD4 attenuates PD-L1 expression in breast cancer [29, 30]. Next, we were interested in the effect of Rg3-CNT on BRD4 and PD-L1 in breast cancer cells. For this purpose, the MDA-MB-231 and BT-549 cells were treated with Rg3 or Rg3-CNT. Interestingly, the treatment of Rg3 failed to affect the expression of PD-L1 in the MDA-MB-231 and BT-549 cells, but the treatment of Rg3-CNT remarkably reduced the expression of PD-L1 in the system (Figure 2A, 2B). Meanwhile, the treatment of Rg3 was not able to inhibit the expression of BRD4 in the MDA-MB-231 and BT-549 cells, while the treatment of Rg3-CNT significantly down-regulated the mRNA and protein levels of BRD4 in the cells (Figure 2C–2F). The similar results were observed in MDA-MB-468 cell lines (Supplementary Figure 2).

### Rg3-CNT decreases the PD-L1 upregulation induced by IFN-γ in TNBC cells

**Figure 3:**
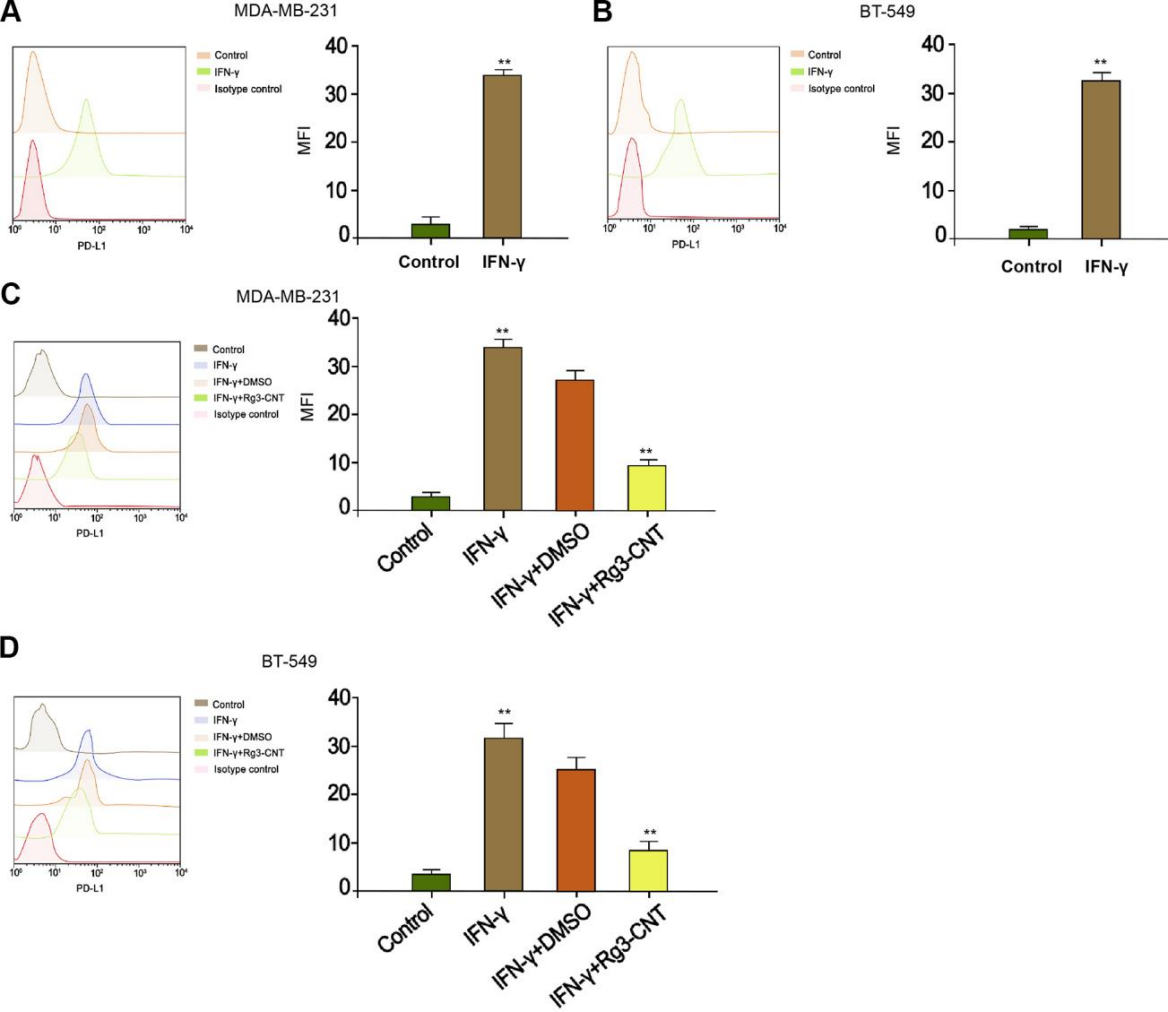
**Rg3-CNT decreases the PD-L1 upregulation induced by IFN-γ in TNBC cells.** (**A**, **B**) The MDA-MB-231 and BT-549 cells were treated with IFN-γ (200 ng/mL). The expression of PD-L1 was analyzed by fluorescence-activated cell sorting (FACS) in the cells. (**C**, **D**) The MDA-MB-231 and BT-549 cells were treated with IFN-γ (200 ng/mL), or co-treated with IFN-γ (200 ng/mL) and Rg3-CNT (60 μg/ml). The expression of PD-L1 was analyzed by fluorescence-activated cell sorting (FACS) in the cells. Data are presented as mean ± SD. Statistic significant differences were indicated: ** *P* < 0.01.

Given that interferon-γ (IFN-γ) was a strong inducer of PD-L1, we further explored the effect of Rg3-CNT on IFN-γ-mediated PD-L1 expression. As expected, the treatment of IFN-γ significantly enhanced the expression of PD-L1 in the MDA-MB-231 and BT-549 cells (Figure 3A, 3B). Meanwhile, the MDA-MB-231 and BT-549 cells were treated with IFN-γ or co-treated with IFN-γ and Rg3-CNT. Significantly, we observed that Rg3-CNT notably reversed the IFN-γ-induced PD-L1 in the cells (Figure 3C, 3D).

### Rg3-CNT represses PD-1 expression

**Figure 4:**
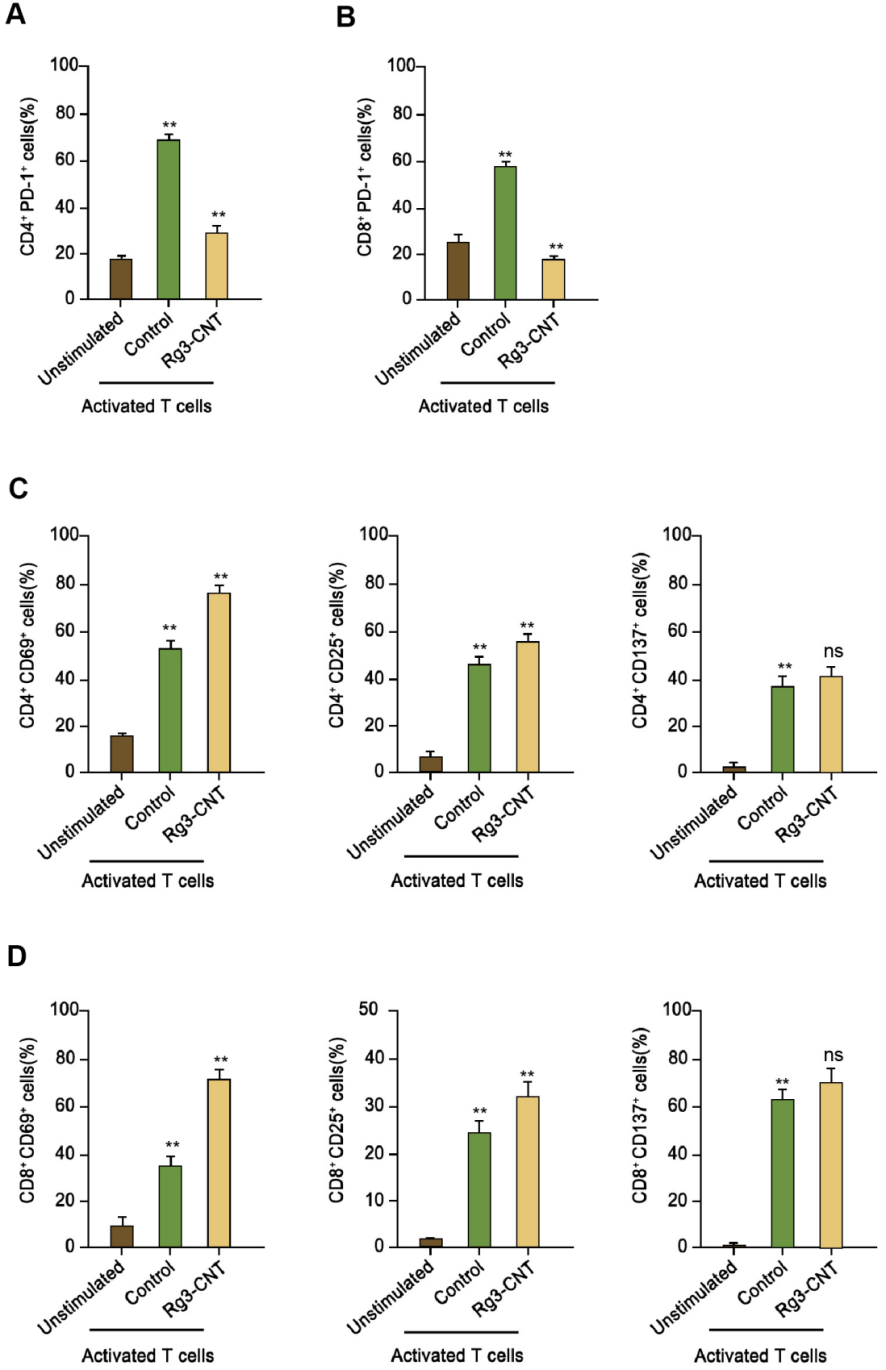
**Rg3-CNT reduces PD-1 expression in activated T cells.** (**A**, **B**) Human PBMCs were cultured, either unstimulated or activated by anti-CD3/CD28 beads, in the presence of control or Rg3-CNT (60 μg/ml) prior to staining with flow cytometry analysis. (**C**, **D**) CD69+, CD25+ and CD137+ percentages were determined among the CD4+ and CD8+ T cell populations. Data are presented as mean ± SD. Statistic significant differences were indicated: ns no significance, ** *P* < 0.01.

Next, we further evaluated the impact of Rg3-CNT on the PD-1 in activated T cells. Remarkably, PD-1 was enhanced in activated T cells (Figure 4A, 4B). Consistently, the treatment of Rg3-CNT significantly stimulated the population of CD4+ and CD69+, CD25+, and CD137+ T cells (Figure 4C). Similarly, the CD8+ and CD69+, CD25+, and CD137+ T cells were induced by treatment of Rg3-CNT in the system (Figure 4D).

### Rg3-CNT suppresses the PD-1/PD-L1 signaling

**Figure 5:**
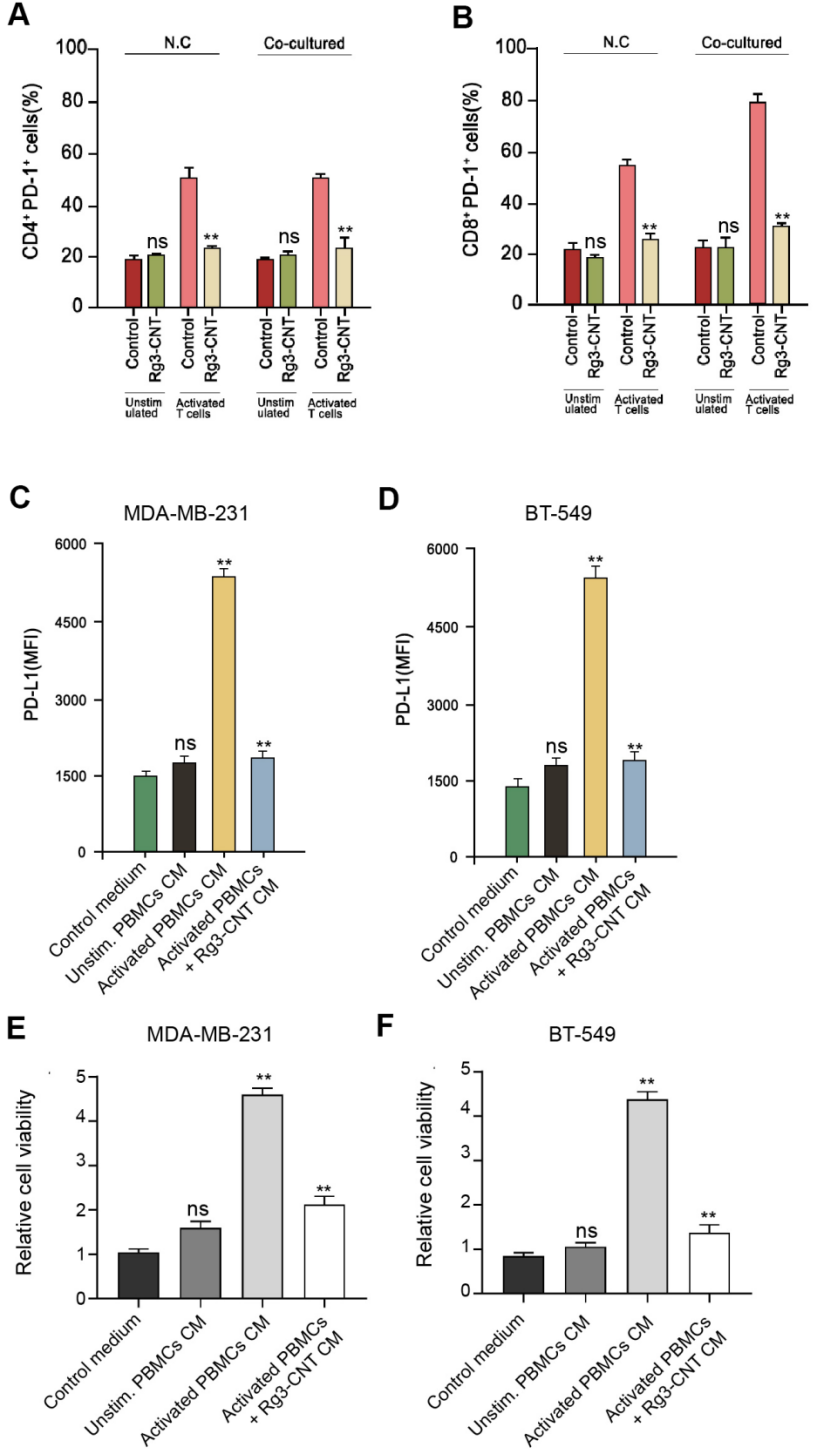
**Rg3-CNT reduces the PD-1/PD-L1 axis in a T cell/triple-negative TNBC cell co-culture system.** (**A**, **B**) MDA-MB-231 or BT-549 cells alone or co-cultured with unstimulated PBMCs or activated T cells obtained from human normal blood donors at a ratio of 1:10 (tumor cells: T cells) were analyzed for PD-L1 expression. (**C**, **D**) MDA-MB-231 and BT-549 cells were cultured with the generated conditioned media prior to PD-L1 expression analysis. (**E**, **F**) The cell viability was measured by the MTT assays in the cells. Data are presented as mean ± SD. Statistic significant differences were indicated: ns no significance, ** *P* < 0.01.

Given that we identified that PD-L1 and PD-1 were both regulated by B Rg3-CNT, we sought to explore the underlying influence by applying the co-culture system of T cell/triple-negative TNBC cells. Significantly, Rg3-CNT reduced PD-1 expression in the activated T cells (Figure 5A, 5B). Moreover, conditioned medium of unstimulated PBMCs failed to regulate PD-L1, while medium of activated PBMCs increased PD-L1 expression in the MDA-MB-231 and BT-549 cells, in which the treatment of Rg3-CNT could significantly block this phenotype in the system (Figure 5C, 5D). In addition, activated PBMCs enhanced the cell proliferation while the treatment of Rg3-CNT was able to reduce the effect in MDA-MB-231 and BT-549 cells (Figure 5E, 5F).

### Rg3-CNT regulates effector T cell interferon-γ secretion

**Figure 6:**
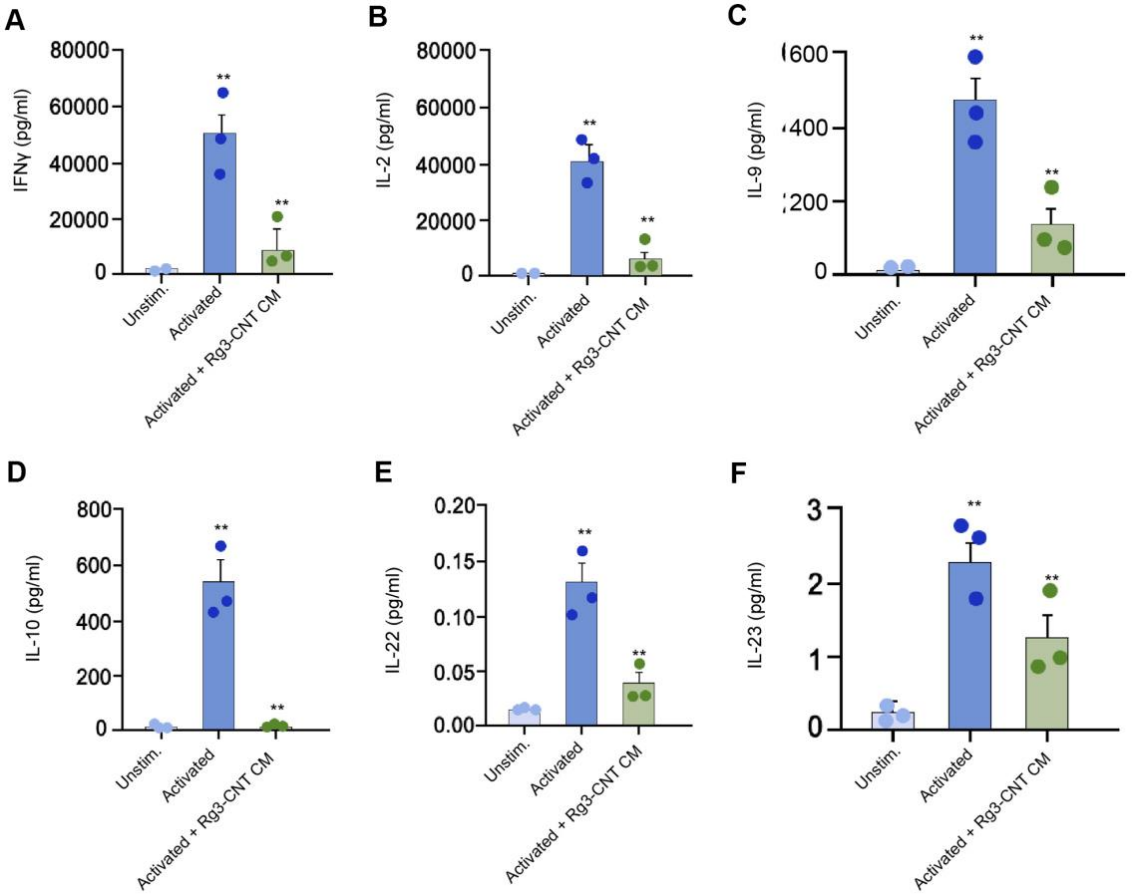
**Rg3-CNT regulates effector T cell interferon-γ secretion.** (**A**–**F**) The levels of the indicated cytokines were analyzed by ELISA assays in either unstimulated or activated PBMCs. Data are presented as mean ± SD. Statistic significant differences were indicated: ** *P* < 0.01.

Furthermore, we found that the levels of IFN-γ, interleukins-2 (IL-2), IL-9, IL-10, IL-22, and IL-23 were stimulated in the activated T cells, in which the treatment of Rg3-CNT was able to reverse this effect in the system (Figure 6A–6F).

### Rg3-CNT suppresses the cell growth of TNBC *in vivo*

**Figure 7:**
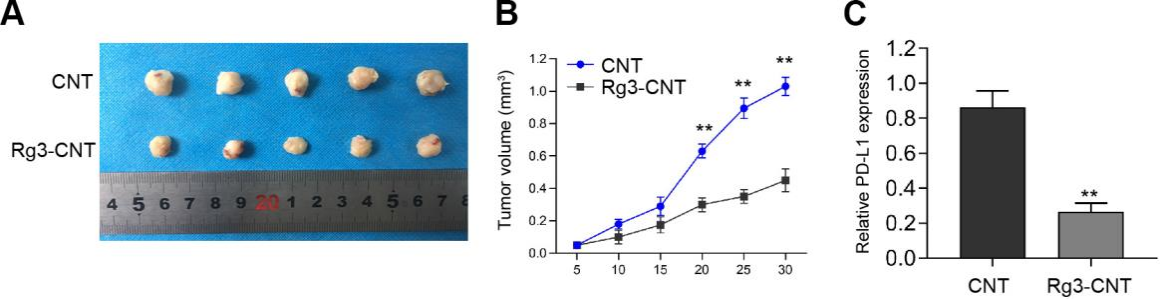
**Rg3-CNT suppresses the cell growth of TNBC *in vivo*.** (**A**–**C**) The nude mice (n = 5) were injected with the MDA-MB-231 cells treated with Rg3-CNT or CNT. (**A**, **B**) Representative images and tumor volume were shown. (**C**) The expression of PD-L1 was detected by qPCR assays in the tumor tissues. ** *P* < 0.01.

We then evaluated the influence of Rg3-CNT on the TNBC cell growth *in vivo*. *Tumorigenicity assays* revealed that Rg3-CNT remarkably attenuated cell growth of TNBC in the nude mice (Figure 7A, 7B). Importantly, the expression of PD-L1 was inhibited by the treatment of Rg3-CNT in the tumor tissues from the mice (Figure 7C).

## DISCUSSION

TNBC is the prevailing malignancy affecting modern women and leading to severe mortality (Woolston, 2015 #10). Carbon nanotubes (CNTs) are advanced nanotechnology with particular properties and structures and have demonstrated a practical role in cancer treatment [14]. Nevertheless, the function of Rg3-CNT in breast cancer pathogenesis remains unclear. In this study, we firstly identified that Rg3-CNT induced the anti-cancer effect on breast cancer by suppressing the PD-1/PD-L1 axis.

Previous studies have found the critical role of CNTs as an important carrier for the treatment of cancers. It has been reported that CNTs induce an inhibitory effect on the viability of malignant glioma cells [31]. CNTs handle the perturbing calcium to promote cancer cell apoptosis *via* low-intensity nanosecond electric pulse [32]. CNTs are able to enhance the sensitivity of breast cancer cells to paclitaxel treatment [33]. CNTs-loaded glycopolymer can target breast cancer [34]. Meanwhile, Rg3 has been well-described as a tumor suppressor of breast cancer. It has been found that ginsenoside Rg3 reduces mesenchymal transition and cancer stemness of breast cancer by regulating myeloid-related suppressor cells [24]. Ginsenoside Rg3 combined with Endostar represses tumor growth of breast cancer cells [35]. Ginsenoside Rg3 suppresses progression of breast cancer by targeting CXCR4 expression [36]. Besides, it has been found that two other gradients of ginsenoside, called Rb1 and Rg1, can be loaded by CNTs and inhibit cancer development [28]. Our results revealed that Rg3-CNT repressed proliferation and increased apoptosis of breast cancer cells. Our data elucidate a new impact of Rg3-CNT on breast cancer progression, providing significant evidence of the function of CNT-loaded anti-cancer treatments in modulating breast cancer. The mechanisms of CNT improving the anti-tumor effect of Rg3 in breast cancer need to be explored in the future. CNT, as a nanoparticle drug carrier, presents several significant properties [16]. CNT may improve the drug-delivery effectiveness of Rg3, which deserves to be investigated in detail.

Targeting PD-1/PD-L1 signaling can reinforce the anti-cancer immunotherapy and attenuates breast cancer progression. It has been reported that Targeting BET protein inhibits the PD-1/PD-L1 signaling enhances anti-cancer immune response in t breast cancer [37]. Small-molecule inhibitor-mediated immune checkpoints blockade targets the PD-1/PD-L1 signaling to stimulate anti-tumor immune response of breast cancer [38]. The administration of PD-1/PD-L1 monoclonal antibody promotes f hypersensitivity reactions in breast cancer [39]. Blockade of TIM-3, PD-L1, and PD-1 regulates cancer-related immune signaling in breast cancer [40]. Our investigation demonstrated that Rg3-CNT attenuated PD-L1 expression and PD-L1 elevation induced by IFN-γ in breast cancer cells. Moreover, Rg3-CNT inhibited PD-1/PD-L1 signaling. These data uncover an unreported correlation of Rg3-CNT with PD-1/PD-L1 signaling and provide new evidence that Rg3-CNT/PD-1/PD-L1 axis for the modulation of breast cancer. Moreover, previous study has shown that Rg3 inhibits PD-L1 expression by regulating Akt and NF-κB in lung cancer [29]. There is a limitation of this study that the mechanisms underlying Rg3-CNT-mediated PD-L1 in TNBC cells are needed to explore in future investigations.

In conclusion, we discovered that Rg3-CNT improved the anti-cancer effect of Rg3 toward TNBC by inhibiting the PD-1/PD-L1 axis. Rg3-CNT may be applied as the therapeutic strategy for the immunotherapy of TNBC.

## MATERIALS AND METHODS

### Cell culture

The MDA-MB-231, MDA-MB-468 and BT-549 cells purchased in ATTCC were cultured in RPMI-1640 (Gibco, USA) with 0.1 mg/mL or 100 units/mL streptomycin/ penicillin (Gibco, USA) and 10% FBS (Gibco, USA), at 37° C and 5% CO2. The transfection in the cells was performed by Liposome 3000 (Invitrogen, USA) according to the manufacturer's instructions.

### Rg3-CNT attachment

Multi-walled CNTs were synthesized and featured as previously reported [28]. Rg3 was purchased (Sigma, USA). CNTs were autoclaved 3 continuous times to eliminate all endotoxin traces. The CNTs oxidation was performed by taking the nanotube of the sulfuric acids and nitric mixture. Functional CNTs were cleaned with purified water numerous times to eliminate any inorganic acid traces, followed by the drying using the vacuum oven. Oxidized CNTs (25 mg) were scattered in distilled water (20 ml) and were attached with Rg3 (5 mg). The mix was sonicated at 0° C for 30 minutes and was stirred for 3 hours at room temperature, followed by centrifuging. The precipitate was collected and the conjugated Rg3-CNTs were dried.

### MTT assays

For MTT assay, MDA-MB-231 and BT-549 cells were digested, collected, and seeded in 96-well plated at a density of 2×104 cells per well and were added to culture medium and incubated for 24 and 48 hours. At indicated time point, MTT reagent was added into each well and incubated for another 4 hours. At last, cell culture medium was discarded and 150 μL DMSO was added to incubate in dark for 15 minutes. The absorbance values at 570nm were detected by a microplate reader (Bio-Tek EL 800, USA).

### Colony formation assays

For colony formation experiment, 1×103 MDA-MB-231 and BT-549 cells were planted in 6-well plates and incubated for two weeks. The visible clones were fixed by methanol, stained with crystal violate for 20 minutes, photographed and counted.

### Flow cytometric analysis

The single-cell suspension was collected and dyed with primary antibodies (BD, USA) for 30 minutes at 4° C. Cell suspension was resuspended using the viability dyes (BD, USA), followed by the flow cytometry analysis. Single-stained and Unstained control were applied for determining background. Data acquisition was carried out using the LSRII flow cytometer (BD, USA) and results were analyzed using FlowJo Software.

### Tumor-reactive T cell priming

T cell from donors' peripheral blood was primed with dendritic cells pulsed by antigens acquired from breast cancer cells in the presence of interleukins 7 (IL-7, 1 ng/mL, Invitrogen) and 2 (IL-2, 10 u/mL, Gibco) for seven days as previously reported [37]. The related cytokines were analyzed by related ELISA assays (Sigma, USA).

### Analysis of cell apoptosis

To detect apoptotic cells, the collected cells were stained by an Annexin V-FITC Apoptosis Detection Kit (CST, USA) following manufacturer’s instruction. In Brief, the MDA-MB-231 and BT-549 cells were stained by Annexin V and PI at room temperature in dark for 15 minutes, subsequently washed with PBS and detected in flow cytometer (BD Biosciences, USA).

### Quantitative reverse transcription-PCR

In brief, total RNAs were isolated by using the Trizol solution (Invitrogen, USA), followed by reverse transcription to cDNAs by an Easy Script kit (TaKaRa, China). The relative expression of miR-181-5p was measured by using a SYBR-Green (Takara, China). The primer sequences: BRD4 F: 5′-GAGCTACCCACAGAAGAAACC-3′; R: 5′-GAGTCGATGCTTGAGTTGTGTT-3′; GAPDH F: 5′-AAGAAGGTGGTGAAGCAGGC-3′, R: 5′-TCCACCACCCAGTTGCTGTA-3′.

### Western blot analysis

Ice-cold RIPA lysis buffer (CST, USA) was added to cells or tumor sections for extraction of total proteins. An equivalent 30 μg protein was separated via a SDS-PAGE and shifted to PVDF membranes. The blots were blocked in 5% non-fat milk for 1 hour, and incubated with primary antibodies against BRD4 (Abcam, USA) and β-actin (Abcam, USA) at 4° C overnight. Next day, the blots were soaked in HRP-conjugated secondary anti-mouse or anti-rabbit antibodies (1:1000, Abcam, USA). Then, an ECL kit (Thermo, USA) was used for the visualization of protein bands in an Odyssey CLx Infrared Imaging System.

### Statistical analysis

All data in this work are provided as means ± SD, and analyzed by Student’s t test or one-way ANOVA method in a GraphPad prism 7 software (Version 19.0). p < 0.05 was considered significant.

## Supplementary Material

Supplementary Figures
