# Supplementary material for: Carbon nanotubes (CNT)-loaded ginsenosides Rb3 suppresses the PD-1/PD-L1 pathway in triple-negative breast cancer
Source: Aging (Albany NY). 2021 Jun 10;13(13):17177–89. doi: 10.18632/aging.203131 (PMC8312428; doi:10.18632/aging.203131)
Supplement: Supplementary Figures [file aging-13-203131-s001.pdf]

SUPPLEMENTARY FIGURES

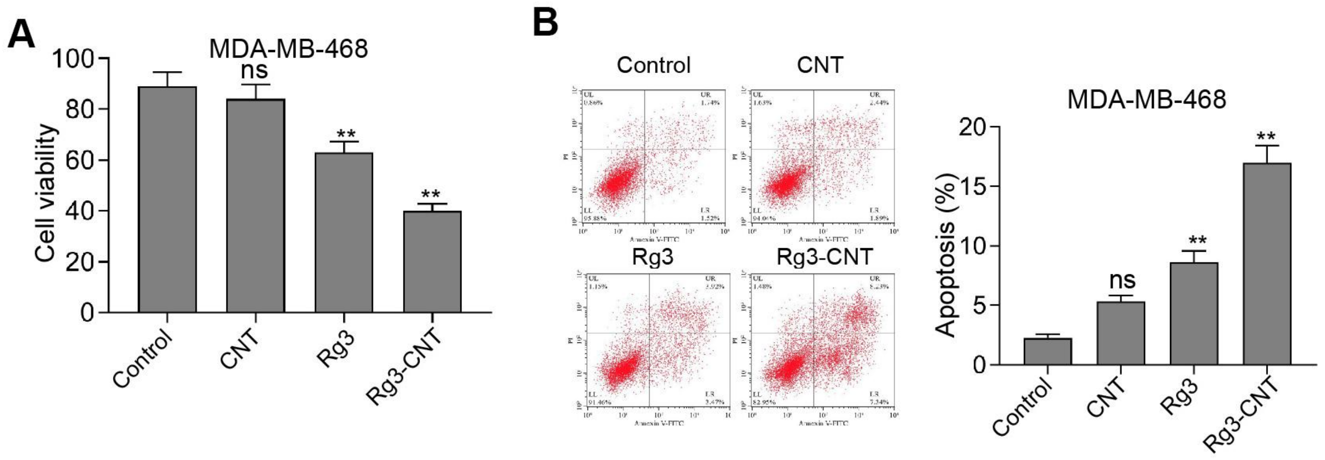

Supplementary Figure 1. Rg3-CNT inhibits proliferation and induces apoptosis of TNBC cells. (**A**, **B**) The MDA-MB-468 cells were treated with CNT, Rg3, or Rg3-CNT. (**A**) The cell viability was measured by the MTT assays in the cells. (**B**) The cell apoptosis was assessed by flow cytometry analysis in the cells. Data are presented as mean ± SD. Statistic significant differences were indicated: ns no significance, ** *P* < 0.01.

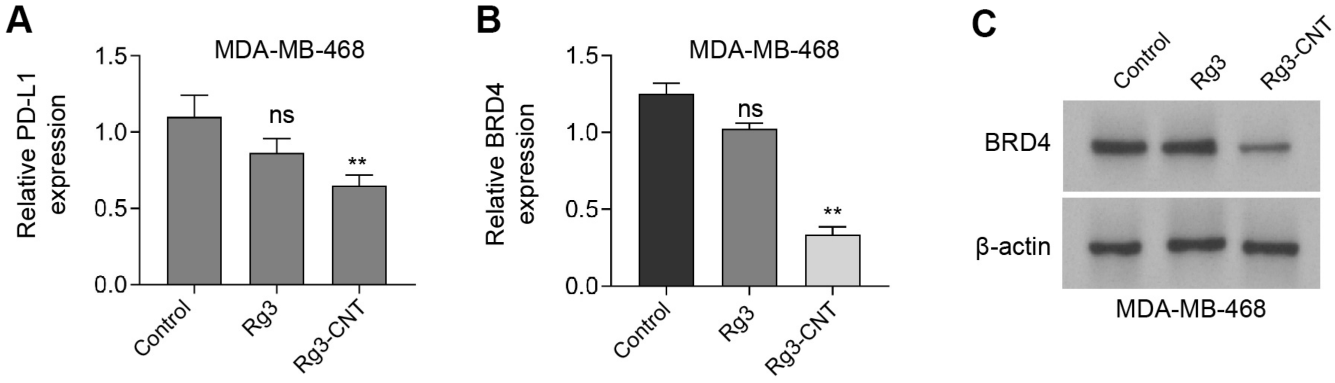

Supplementary Figure 2. Rg3-CNT decreases PD-L1 expression in TNBC cells. (**A**–**C**) The MDA-MB-468 cells were treated with Rg3 (60 μg/ml) or Rg3-CNT (60 μg/ml). (**A**) The expression of PD-L1 was analyzed by fluorescence-activated cell sorting (FACS) in the cells. (**B**) The mRNA expression of BRD4 was measured by qPCR in the cells. (**C**) The protein expression of BRD4 was tested by Western blot analysis in the cells. Data are presented as mean ± SD. Statistic significant differences were indicated: ns no significance, ** *P* < 0.01.
